# Quantifying *Cunninghamia lanceolata* Foliar Water Uptake and Reverse Transport Provides a New Approach to Improving Drought Tolerance

**DOI:** 10.1002/ece3.72953

**Published:** 2026-01-20

**Authors:** Ting Xiang, Jianbo Jia, Bo Han, Chenhui Zhang, Wende Yan

**Affiliations:** ^1^ Central South University of Forestry and Technology Changsha China; ^2^ Key Laboratory of Soil and Water Conservation and Desertification Combating Ministry of Education Changsha China; ^3^ Hunan Lutou Forest Ecosystem Orientation Observation and Research Station Yueyang China

**Keywords:** *Cunninghamia lanceolata*, drought stress, foliar water uptake, isotope tracing, reverse transport

## Abstract

Foliar water uptake (FWU), an important source of supplemental water for plants, provides a novel pathway for alleviating drought stress. However, quantitative analysis of reverse water transport after water uptake in plant leaves has been insufficient, which has become a bottleneck in the study of adaptive plant survival under drought stress. This study investigates *Cunninghamia lanceolata* ( *C. lanceolata* ) using pot experiments with controlled watering, simulated fog environments, and stable isotope techniques to quantitatively explore the conditions that facilitate water absorption in *C. lanceolata* seedlings under drought stress, the thresholds for reverse water movement in various organs, and whether FWU enhances drought resistance. The results indicated that FWU occurred when the soil water content (SWC) fell below 60% of field capacity for 2 h in a foggy water environment. After 12 h of fog water treatment, the leaf water potential (LWP) and leaf water content (LWC) of *C. lanceolata* seedlings significantly improved under drought stress. When SWC exceeded 60% of field capacity, retrograde transport did not occur. When SWC ranged between 45% and 60% of field capacity, leaf water uptake retrograde transport to the stem, resulting in an increase in *δ*_*D*_ value by 20.54‰ ± 5.16‰. When SWC dropped between 30% and 45% of field capacity, retrograde transport to the rhizosphere soil occurred, with *δ*_*D*_ values increasing by 30.94‰ ± 1.4‰. Water absorbed by leaves can move along the leaf‐stem‐root water potential gradient into the xylem and surrounding soil, with maximum utilization rates of 16.26%, 11.13%, and 1.66%, respectively, thereby improving the plant's water status. From the above, it can be seen that *C. lanceolata* can effectively alleviate drought stress through leaf water uptake, and the reverse water transport threshold can be used as a research basis to provide new ideas for plants to cope with drought stress.

## Graphical abstract

These findings demonstrate that *C. lanceolata* can effectively alleviate drought stress by absorbing fog water through their leaves. Additionally, the identified water threshold for reverse water transport provides a foundation for future research, offering new insights into plant strategies for coping with drought stress.

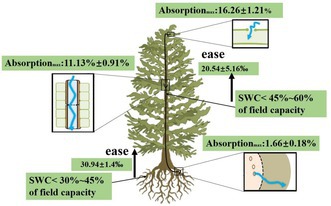

## 1 Introduction

Global climate change alters precipitation patterns, causing increased variability in both space and time. This leads to more frequent, intense, and prolonged drought events, which pose significant threats to forest ecosystems (Chen et al. 2025; Huang et al. 2025; Yuan et al. 2023). Recent research indicates a link between high temperatures, drought, and increased vegetation mortality across different global ecosystems and plant functional types (Hartmann et al. 2022; Rattan et al. 2025; Sun et al. 2025), and many studies believe that drought is the main cause of tree death (Sterck et al. 2024; Wang et al. 2021). Research indicates that the death of woody plants due to drought is not confined to arid regions; it also frequently occurs in well‐watered temperate, tropical, and subtropical forests (Bauman et al. 2022; Caminero et al. 2018; Ziegler et al. 2024). Historically, research on plant water sources in long‐term arid forest environments has largely focused on root systems for water absorption (Li et al. 2024; Serret et al. 2020). Few studies have considered the reverse process, where leaves can absorb water from their surfaces and transfer it to branches and roots.

Foliar water uptake (FWU) is considered to be a mechanism by which plants can obtain water from the atmosphere through leaves (Guzmán‐Delgado et al. 2021; Schaepdryver et al. 2022). Early studies primarily concentrated on water interception and evaporation from plant leaves (Wang and Guo 2024; Xu et al. 2022), often overlooking the direct absorption of atmospheric moisture, such as fog water, by leaves. Fog water is prevalent in forests worldwide and serves as a crucial water source for ecosystems. It significantly enhances forest water conservation and impacts the overall water balance (Templer et al. 2015). In regions with frequent fog, vegetation can thrive by absorbing this moisture (Henschel and Seely 2008). Additionally, in areas with limited rainfall, fog water becomes an essential water source (Yang et al. 2024). Some plants adapt their leaf characteristics to enhance the interception and absorption of atmospheric moisture. When water is predominantly present in the form of fog or dew, it condenses on leaf surfaces and is directly absorbed through structures like cuticles and trichomes (Berry et al. 2019; Singh and Pandey 2025).

Recent advancements in technologies such as isotope tracers, stem sap flow measurements, and water potential assessments offer new opportunities to explore the transmission pathways of fog water within ecosystems, as well as the processes of water and nutrient cycling (Eller et al. 2016). By utilizing this technology, researchers have confirmed that fog water acts as a significant source of moisture for key plant species in typical arid regions of Saudi Arabia (Valjarević et al. 2023), as well as for deciduous shrubs in the arid area of Santa Barbara (Emery 2016). In various ecosystems, leaves play a crucial role in enhancing the water status of plants during drought conditions by absorbing fog water (Kagawa 2022; Singh and Pandey 2025). However, there remains a gap in experimental evidence regarding the ability of trees to absorb fog water through their leaves, as well as the effects of fog water on plant physiological conditions during the distinct dry and wet seasons in tropical Asia.

*Cunninghamia lanceolata* has a broad geographic distribution across the subtropical and tropical margins, as well as the southern edge of the warm‐temperate zone in China. The area of *C. lanceolata* plantation forests in China spans approximately 11 million hectares (ha), accounting for about 12.9% of the country's total plantation forests (SFAPRC 2015; Wu et al. 2020). However, in recent years, global warming has led to seasonal droughts during the summer and fall in the *C. lanceolata* distribution areas of China's subtropical monsoon climate zone (Li et al. 2023; Ren et al. 2024). These droughts often subject *C. lanceolata* trees to drought stress (Gao et al. 2021; Lei et al. 2023), threatening the growth of *C. lanceolata* seedlings and young forests (Li et al. 2020; Villagra et al. 2013). Consequently, studying the water supplementation of *C. lanceolata* during the dry season is particularly important. Fog water serves as a crucial means of water supplementation, providing essential insights into the drought resistance of *C. lanceolata* forests under drought stress (Fu et al. 2016).

Building on this foundation, the present study focuses on *C. lanceolata* seedlings as the research subjects and employs stable isotope labeling of fog water to conduct simulation experiments. By controlling various environmental variables and utilizing the isotope labeling technique, this research aims to explore the dynamics of water reverse migration following absorption through the leaves of *C. lanceolata* . The study will quantify the utilization ratios of reverse migration heavy water in the leaves, branches, and rhizosphere soil, while also investigating the conditions under which *C. lanceolata* seedlings can effectively absorb fog water. Additionally, it will identify the threshold values of reverse migration water in different plant organs and assess whether this mechanism can enhance the drought resistance of the seedlings. The findings of this research are expected to provide valuable theoretical and practical insights into the water absorption processes of *C. lanceolata* leaves and their adaptive mechanisms to soil drought conditions.

## 2 Materials and Methods

### 2.1 Study Site

**FIGURE 1:**
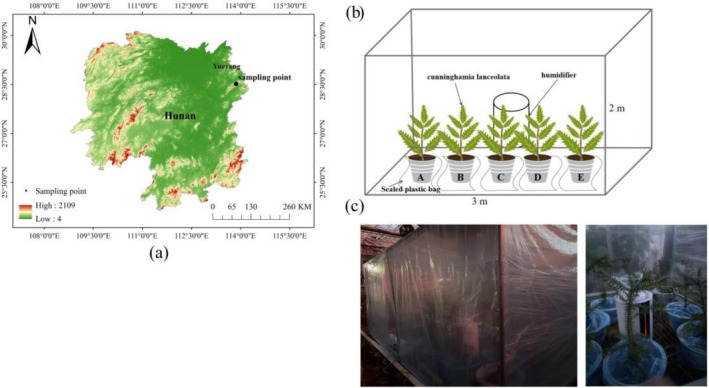
Research point location and self‐made experimental device for fog and water environment simulation. (a) Location of the positional observatory and research station of the Lutou Forest Ecosystem, Hunan Province, China; (b) schematic diagram of simulated fog‐water environment; (c) actual diagram.

The study area is located in the greenhouse of the Positional Observation and Research Station of the Lutou Forest Ecosystem in Hunan Province (28°31′7″ N–28°38′ N, 113°51′52″ E–113°58′24″ E) (Figure 1a). The study area experiences a typical subtropical monsoon climate, with an average annual temperature of 18.5°C and an average annual precipitation of 1450.8 mm. The rainy season lasted from April to September, accounting for 72.4% of the total precipitation, while the dry season occurred from October to March. The understory vegetation is mainly composed of tree species such as *Corylopsis sinensis Hemsl*, *Ilex chinensis Sims*, and *Asparagus cochinchinensis (Lour.) Merr*.

### 2.2 Experimental Design

#### 2.2.1 Potted Plant Water Control Experiment

The experimental materials consisted of 2‐year‐old superior *C. lanceolata* seedlings, artificially propagated at the Lutou Forest Farm nursery base in Hunan Province, with a total of 250 plants. Each seedling had an average mass of 125 g and an average height of 60 cm. The seedlings were transplanted into flower pots measuring 30 cm in diameter and 28 cm in height for cultivation. For the first 6 months, the seedlings were adequately watered daily at 18:00 to ensure stable growth.

Subsequently, 200 pots of *C. lanceolata* seedlings were selected for water control treatment. During this phase, the seedlings were watered daily (*δ*_*D*_ = −21.65‰ ± 2.12‰) to maintain the soil water content (SWC) within the prescribed range for each treatment. A pre‐experiment was conducted using 10 pots of *C. lanceolata* seedlings to determine their permanent wilting point, defined as the SWC for a given soil type at which the plants undergo permanent wilting. The results indicated that the permanent wilting point of *C. lanceolata* in the selected soil ranged from 5.5% to 5.8%. This finding suggests that when the effective water content falls below this range, the growth of *C. lanceolata* seedlings ceases. Therefore, the effective range for controlling SWC in this experiment was 5.5%–29%. Drought stress levels were set in a stepwise manner, with SWC decreasing incrementally by gradients corresponding to 15%–20% of field water capacity (the field water capacity in this experiment was 29%). Accordingly, combined with the relevant reference and actual conditions (Huber et al. 2023; Li et al. 2024), a total of five soil water gradients were established for this study (Table 1), with 30 pots in each group. During the watering control phase, soil moisture content was continuously monitored in real‐time using the EM50/R data logger (Decagon, USA). Regular replenishment was performed daily to maintain soil moisture within the specified range (Table 1). The soil moisture gradients were established based on previous research and the specific conditions of the experiment. The selection of each range is informed by studies on plant physiological adaptability. Moisture range measure from 15% to 30%: in this range, *C. lanceolata* may exhibit significant water stress symptoms, representing extreme drought conditions. This range is suitable for studying physiological responses under severe water scarcity, such as wilting and early mortality. Moisture range from 30% to 45% moisture range: although water remains insufficient, plants may maintain some growth by adjusting their physiological mechanisms. This stage is appropriate for observing the adaptability of *C. lanceolata* under moderate water stress. Moisture range from 45% to 60% moisture range: this range offers relatively suitable growing conditions, providing some water supply. It is suitable for examining the growth performance and physiological changes of *C. lanceolata* under light drought stress. Moisture range from 60% to 80%: this range represents optimal conditions for growth, with relatively ample moisture. It is ideal for researching the physiological performance and growth potential of *C. lanceolata* under favorable conditions. Moisture range from 80% to 100% moisture range: while this range is beneficial for growth, excessive moisture can lead to root anoxia, adversely affecting plant health.

**TABLE 1:** SWC of the five treatments in the control experiment.

Group	Range of proportions of field water holding capacity (%)	Actual soil water content range (%)	Soil moisture status
A	15–30	5.5–9	Severe drought
B	30–45	9–13	Moderate drought
C	45–60	13–17	Light drought
D	60–80	17–23	Natural Suitability
E	80–100	23–29	Abundant water

*Note:* The field capacity is based on 29.0%.

After this control phase, we conducted simulated fog water environment experiments with heavy water isotope labeling. The table below presents the soil moisture characteristics of Groups A‐E before the simulated fog environment experiment. As all potted plants were sealed with transparent plastic bags on their pots and stems during the fog simulation, there was no additional water input into the soil during the simulation. Therefore, we focused on the soil water potential and moisture content characteristics of Groups A–E before the fog environment simulation (Table 2).

**TABLE 2:** Changes in soil moisture and water potential.

Group	A	B	C	D	E
Soil moisture (%)	6.78 ± 0.58	10.71 ± 1.23	13.53 ± 1.46	18.84 ± 1.17	24.22 ± 2.04
Soil water potential (MPa)	−1.41 ± 0.27	−1.18 ± 0.14	−0.76 ± 0.11	−0.31 ± 0.05	−0.16 ± 0.01

*Note:* A, B, C, D, and E indicate the treatments with an SWC of 5.5%–9%, 9%–13%, 13%–17%, 17%–23%, and 23%–29%, respectively.

#### 2.2.2 Fog Water Environment Device Setup

The fog‐water environment in the experiment was simulated in a dark room using a humidifier (Figure 1b). This timing was designed to align with natural fog and dew conditions. Since fog typically occurs at night and early morning, and based on research on the timing of FWU in subtropical monsoon climate zones (Wenping et al. 2021), each fog simulation period was scheduled from 18:00 to 6:00 the following day. Before the water control experiment, a pre‐experiment was conducted using 40 pots of *C. lanceolata* seedlings to determine the simulation period. The results showed that leaf water uptake increased after 2 h of exposure to fog water treatment compared to pretreatment levels. As time progressed, humidity continued to rise, and water uptake gradually increased. At 4 h, water uptake significantly increased; at 6 h, it rose further but did not reach saturation. After 12 h, leaf water absorption approached saturation and stabilized. Therefore, four fog environment simulation periods were established: 2 h (18:00–20:00), 4 h (18:00–22:00), 6 h (18:00–00:00), and 12 h (18:00–6:00). During each fog‐water simulation period, changes in temperature and humidity were recorded both indoors and outdoors. The results indicated that the patterns of temperature and humidity changes were generally consistent across all experiments. During the experimental period, the relative humidity in the dark room was maintained at 90%–100%, while the temperature difference between the inside and outside of the dark room was less than 1°C. This minimal temperature difference suggests that its impact on the experimental results could be considered negligible.

#### 2.2.3 Isotope Labeling and Tracing Experiments

When the SWC stabilized within the specified range for each of the five groups, two treatments were applied to each group: 40 *C. lanceolata* seedlings were labeled with nondistilled water (*δ*_*D*_ = −21.65‰ ± 2.12‰) as the control group CK, and another 20 were labeled with heavy water (*δ*_*D*_ = 495‰ ± 1.98‰) and set as the treatment group EG (Table 3). Before the simulated fog‐water environment test, the edges of the pots and the trunks of the seedlings were sealed with transparent plastic bags to prevent precipitation from entering the soil. Additionally, the potting soil was made waterproof to ensure the integrity of the experiment (Figure 1c). Subsequently, 3–4 individual *C. lanceolata* plants were randomly selected from each treatment for simulated mist experiments with durations of 2 h (18:00–20:00), as well as for control treatments without mist simulation. Samples were collected from the leaves, branches, and soil near the root zone before each isotope labeling experiment began, along with measurements of leaf moisture content (LMC) and leaf water potential (LWP). After completing the 2‐h mist simulation isotope labeling experiment, the procedure was repeated with a random selection of another 3–4 *C. lanceolata* plants for simulations lasting 4, 6, and 12 h.

**TABLE 3:** Orthogonal experimental treatment of CK and EG by the isotope labeling method.

Treatment	CK				EG			
A	A_2 dw_	A_4 dw_	A_6 dw_	A_12 dw_	A_2 hw_	A_4 hw_	A_6 hw_	A_12 hw_
B	B_2 dw_	B_4 dw_	B_6 dw_	B_12 dw_	B_2 hw_	B_4 hw_	B_6 hw_	B_12 hw_
C	C_2 dw_	C_4 dw_	C_6 dw_	C_12 dw_	C_2 hw_	C_4 hw_	C_6 hw_	C_12 hw_
D	D_2 dw_	D_4 dw_	D_6 dw_	D_12 dw_	D_2 hw_	D_4 hw_	D_6 hw_	D_12 hw_
E	E_2 dw_	E_4 dw_	E_6 dw_	E_12 dw_	E_2 hw_	E_4 hw_	E_6 hw_	E_12 hw_

*Note:* CK stands labeled with nondistilled water, and EG stands labeled with heavy water. A, B, C, D, and E represent the treatments with an SWC of 5.5%–9%, 9%–13%, 13%–17%, 17%–23%, and 23%–29%, respectively. 2, 4, 6, and 12 represent the treatment of simulated fog‐water environment for 2, 4, 6, and 12 h (1200 mL/h). dw and hw nondistilled water labeling and heavy water labeling.

#### 2.2.4 Monitoring of Meteorological Factors and Soil Moisture Content and Water Potential

During the simulation of a foggy environment, a small hand‐held Kestrel weather station (NK5500, OnsetComputer Corp., USA) was used and set up inside and outside the darkroom. The device can monitor temperature (Ta, °C), relative humidity (RH, %), wind speed (*U*), and other conventional meteorological data in real time, and the frequency of data acquisition is set to 15 min/time. The monitoring of soil water potential and water content was conducted by an EM50 soil moisture monitoring system, so that the SWC of each group was stable within its preset range.

### 2.3 Isotope Sample Collection and Isotope Composition Determination

After the simulated fog‐water environment experiment, samples were collected from leaves, stems, and rhizosphere soil. Sampling was conducted 2 h after the conclusion of the experiment to account for the time lag in water migration following leaf water absorption. During sampling, moisture on the leaf surface was wiped dry to prevent interference with the experimental analysis. For branch collection, 3–5 cm sections of lignified branches were cut using branch clippers. The bark was removed, leaving only the woody tissue for analysis. For soil sampling near the roots, smaller soil particles were prioritized. The collected leaf and branch samples were divided into three parts for analysis. One portion was sealed in an aluminum box and dried at 105°C to determine the leaf water content (LWC). Another portion was sealed in a clean 50 mL polyethylene bottle, and the LWP was measured using a PMS pressure chamber (PMS Inc., USA). The final portion was placed in a 10 mL centrifuge tube, sealed with paraffin film, and immediately stored at −20°C until isotope analysis. Similarly, three pots of *C. lanceolata* seedlings were randomly selected from groups A to E for the 4‐, 6‐, and 12‐h simulated fog‐water environment experiments. After completing the experiments for the CK (*δ*_*D*_ = −21.65‰ ± 2.12‰), the EG was tested under the simulated fog‐water environment with an isotope ratio of approximately (*δ*_*D*_ = 495‰ ± 1.98‰). The experimental procedures and sampling methods for the EG group were consistent with those used for the CK group.

### 2.4 LWP Measurement

Branches with intact leaves were collected and placed in black plastic bags for transport to the laboratory. The branches were then allowed to lose moisture under natural conditions, creating a gradient of LWP. A single leaf was selected from each branch, and its LWP (𝜓0, MPa) was measured using a pressure chamber (Plant Moisture Stress, Corvallis, Oregon, USA).

### 2.5 Isotope Sample Determination

In nature, the ratio of heavy to light isotopes (fractionation coefficient) is quite small. Therefore, the isotopic composition of water is typically expressed as a per mille difference relative to Standard Mean Ocean Water (SMOW). When *δ*_*D*_ is positive, it indicates that the sample is enriched in deuterium (D) compared to SMOW. Conversely, when *δ*_*D*_ is negative, it signifies a depletion of the isotope in the sample relative to the SMOW standard. The isotope samples were analyzed using a fully automatic vacuum condensation extraction system (Li‐2100, LICA, China) to extract water from the collected samples. The *δ*_*D*_ isotope values in the water samples were then measured using a laser water isotope analyzer (DLT‐100, ABB, USA). The *δ*_*D*_ value in the water sample measured was the thousandth difference from the “SMOW,” and the determination accuracy was ±0.31‰, and the formula was as follows:

(1) $$\delta_{X}=\left(\frac{R_{\text{sample}}}{R_{\text{standard}}}-1\right)\times 1000$$

where *δ*_*X*_ represents *δ*_*D*_; *R*_sample_ and *R*_standard_ represent the isotopic content of *D* in the sample and standard average ocean water, respectively. The unit of *δ*_*X*_ is per mille‰.

The presence of labeled isotopes in the leaves, branches, and rhizosphere soil of *C. lanceolata* indicates that *C. lanceolata* has leaf water absorption or reverse water migration. Based on the isotope conservation principle, a binary linear mixed model was used to estimate the utilization ratio of heavy water absorbed by leaves, stems, and rhizosphere soil (Phillips and Gregg 2003). The formula is as follows:

(2) $$\delta_{t}=f_{A}\times \delta_{A}+\left(1-f_{A}\right)\times \delta_{B}$$

(3) $$f_{A}=\left(\frac{\delta_{t}-\delta_{B}}{\delta_{A}-\delta_{B}}\right)\times 100\%$$

where: *δ*_*t*_: *δ*_*D*_ value measured in leaf‐stem‐rhizosphere soil; *δ*_*A*_: *δ*_*D*_ value of labeled heavy water; *δ*_*B*_: *δ*_*D*_ value of distilled water; *f*_*A*_: utilization ratio of heavy water.

### 2.6 Data Analysis

All data were recorded using Microsoft Excel 2009 and analyzed using SPSS 22.0. Independent‐sample *t*‐tests were used to examine differences in LWP and LWC before and after exposure to the simulated fogging environment. One‐way ANOVA was employed to compare isotope values between the EG and CK groups, while two‐way ANOVA was used to analyze differences in the hydraulic traits of cedar trees under varying SWCs and fogging durations. All data were assessed for normality and subjected to ANOVA chi‐square analysis. Data in the graphs are presented as mean ± standard deviation. Histograms were visualized using the “ggplot2” package (Villanueva and Chen 2019), sample plots were generated using ArcGIS 10.2, and all other graphs were created with Origin 2021.

## 3 Results

### 3.1 Leaf Water Content and Leaf Water Potential Changes

**FIGURE 2:**
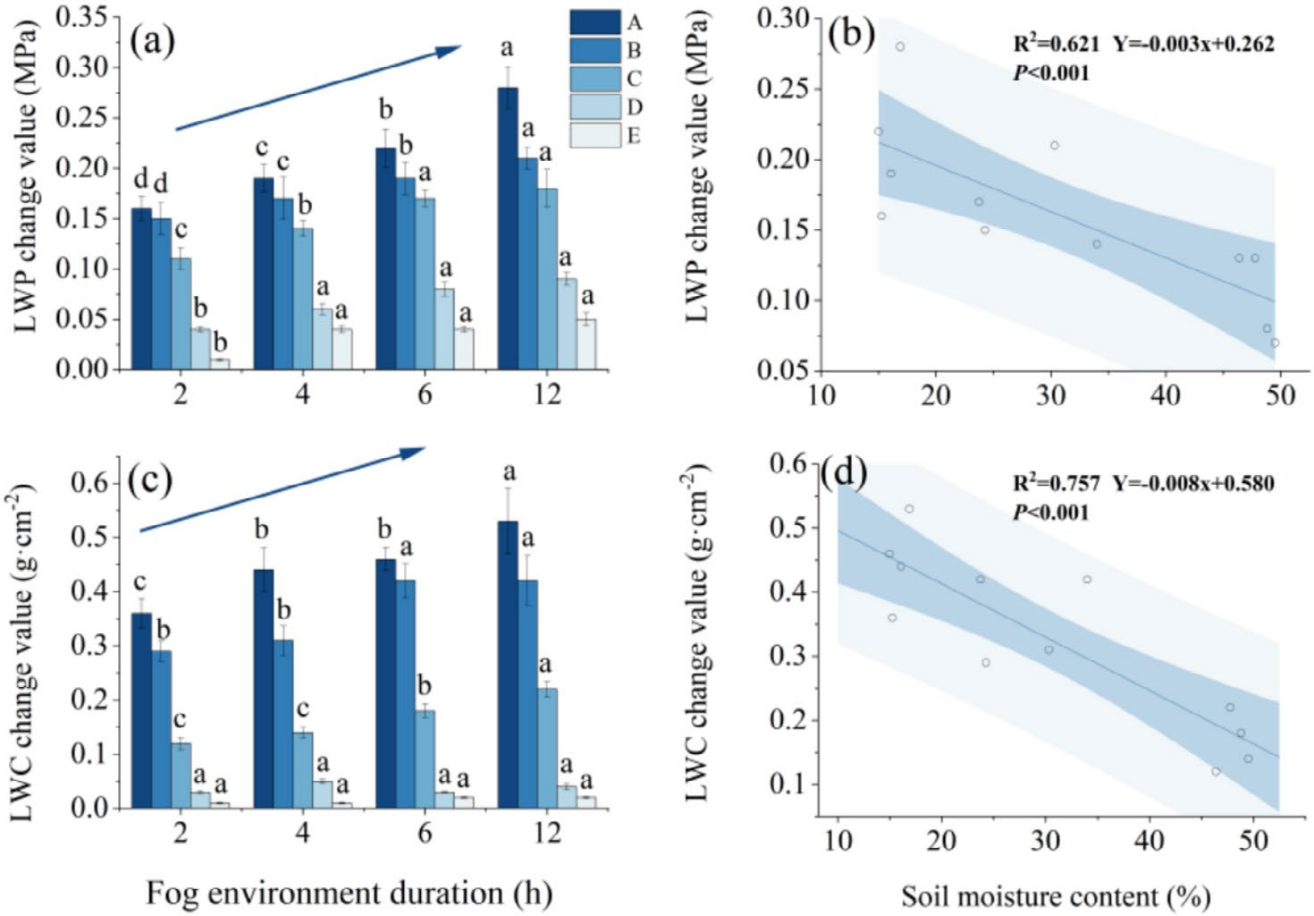
Variation in leaf water content and leaf water potential. (a, c) Changes in leaf water potential and leaf water uptake as differences before and after absorption, with blue arrows indicating trends related to fog duration (2, 4, 6, and 12 h). Lower‐case letters indicate significant differences under similar drought stress. (b, d) Variations in leaf water potential and uptake in response to changes in soil water content.

In the potting control experiments, under a 2–12 h fog‐water environment, significant differences (*p* < 0.05) in the changes in LWP and LWC were observed between groups A and C. No significant differences were found between the treatments in groups D and E (Figure 2a,c). This suggests that leaf water uptake occurs after 2 h of exposure to the foggy water environment, provided that the SWC is below 60% of the field capacity. In groups A–C, SWC, duration of exposure to the fog water environment, and their interaction significantly influenced the changes in leaf water uptake and LWP (*p* < 0.01) (Table 4). The changes in LWP and the amount of change in LWC increased with time in the fog‐water environment and were negatively correlated with SWC, decreasing as SWC increased (Figure 2b,d). In the 12 h fog‐water environment, the changes in LWP and LWC for treatments A, B, and C were as follows: A (0.28 MPa; 0.53 g·cm^−2^), B (0.21 MPa; 0.42 g·cm^−2^), and C (0.18 MPa; 0.22 g·cm^−2^). The greatest changes were observed in treatment A. By quantifying the differences in LWP and LWC changes under varying drought stresses, it was found that LWP and LWC increased by 35% and 43%, respectively, significantly enhancing the leaves' water absorption capacity.

**TABLE 4:** Two‐way ANOVA of leaf water uptake and leaf water potential changes under different soil moisture levels and fog water environment durations.

Treatment	Response variable	*F* value	df	*p*
SWC	LWC change value	1721.47	2	< 0.01
	LWP change value	716.419	2	< 0.01
The duration of the fog water environment	LWC change value	219.733	3	< 0.01
	LWP change value	92.622	3	< 0.01
Interaction	LWC change value	12.898	6	< 0.01
	LWP change value	92.435	6	< 0.01

### 3.2 Characterization of Isotopic Variations Across Different Plant Organs

**FIGURE 3:**
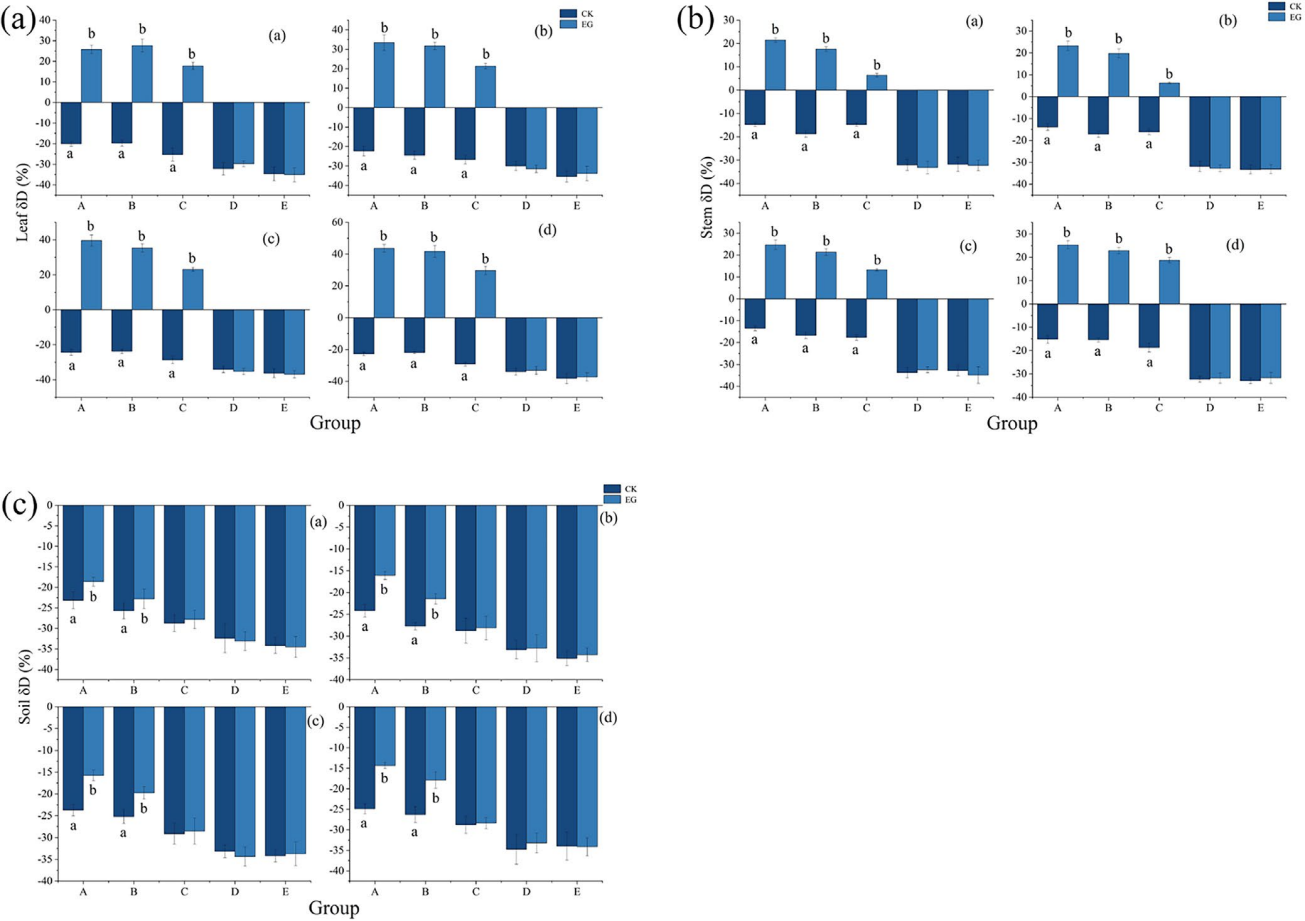
Mean changes in δD values of *C. lanceolata* leaf (a), stem (b), and rhizosphere soil (c). EG and CK represent the experimental and control groups, respectively. Different lowercase letters indicate significant differences (*p* < 0.05) between EG and CK under the same fog duration and drought stress.

Overall, the *δ*_*D*_ values in the EG group gradually increased with the duration of the simulated fog‐water environment (Figure 3). Under different drought stress conditions, the *δ*_*D*_ values of leaf and stem water differed significantly (*p* < 0.05) between the CK and EG groups in treatments A–C (SWC ≤ 60%). In the A treatment, under the simulated fog water environment lasting 2–12 h, the *δ*_*D*_ values of the CK and EG groups reached their maximum after 12 h, at 43.66‰ and 25.32‰, respectively. The *δ*_*D*_ values decreased with increasing SWC. The average difference in *δ*_*D*_ values between the CK and EG groups for leaf water and stem across the A–C groups was 20.54%. In the D and E treatments, no significant difference (*p* > 0.05) was observed in the *δ*_*D*_ values of leaf and branch moisture between the EG and CK groups, indicating no enrichment of heavy water (Figure 3). By quantifying the difference in water uptake from leaves to stems at soil moisture contents below 45%–60% of field capacity, it can be concluded that leaf water uptake reverses the transport of water to the stems, resulting in an increase of 20.54‰ ± 5.16‰ in *δ*_*D*_ values (Table 5).

**TABLE 5:** The change of *δ*_*D*_ value of continuous leaf‐stem and stem‐rhizosphere soil migration in a simulated fog environment.

Reverse migration	Treatment	2 h (‰)	4 h (‰)	6 h (‰)	12 h (‰)	Mean value (‰)
Leaf‐stem	A	9.72 ± 2.04	18.63 ± 3.82	25.64 ± 2.12	25.88 ± 1.45	20.54 ± 5.16
	B	11 ± 4.93	19.35 ± 2.9	21.07 ± 4.25	25.34 ± 3.9	
	C	22.02 ± 1.77	25.61 ± 3.84	20.92 ± 1.32	21.3 ± 3.15	
Stem‐rhizosphere soil	A	31.62 ± 2.19	29.11 ± 3.06	30.23 ± 0.92	29.92 ± 4.84	30.94 ± 1.4
	B	33.45 ± 2.66	30.65 ± 1.38	32.62 ± 5.03	29.89 ± 4.26	

*Note:* A, B, and C represent the treatments with an SWC of 5.5%–9%, 9%–13%, and 13%–17%, respectively.

The difference in *δ*_*D*_ values of rhizosphere soil water was significant (*p* < 0.05) in treatments in groups A to B (SWC ≤ W45%) and increased with the duration of the simulated fog environment. In treatment A, the rhizosphere soil water *δ*_*D*_ value reached a maximum value of −14.32‰ after 12 h of simulated fog‐water environment, and the average *δ*_*D*_ values of stem and rhizosphere soil sections of group A–B treatments differed by 30.94% between group CK and EG. There was almost no significant difference in the *δ*_*D*_ value of rhizosphere soil between the EG and CK groups (*p* > 0.05). By quantifying the difference in stem to rhizosphere soil at soil moisture contents below 30%–45% of field water holding capacity, it can be concluded that leaf water uptake reverses the transport of water to the stems, resulting in an increase of 30.94‰ ± 1.4‰ in *δ*_*D*_ values (Table 5).

### 3.3 Proportion of Reverse Transport by Organ

**FIGURE 4:**
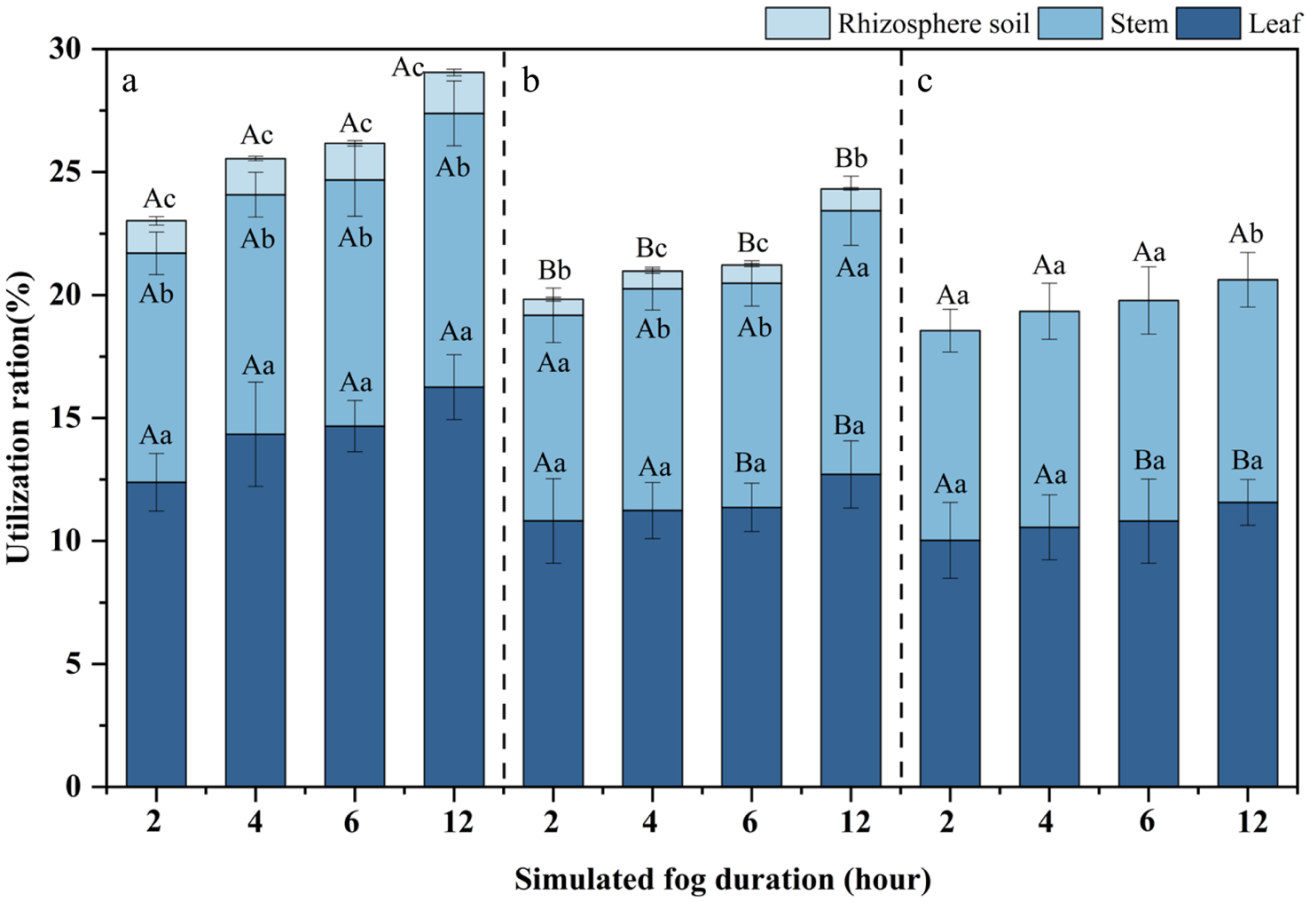
Utilization of fog water by *C. lanceolata* leaf, stem, and rhizosphere soil. (a), (b) and (c) represent the treatments with an SWC of 5.5%–9%, 9%–13%, and 13%–17%, respectively. Different lowercase letters indicate significant differences (*p* < 0.05) in fog water utilization rates among different plant parts under the same drought stress and fog duration. Different uppercase letters indicate significant differences (*p* < 0.05) in fog water utilization rates at the same plant part under varying drought stresses and the same fog duration.

As shown in Section 3.1, since leaf absorption of fog water did not occur in groups D–E, the proportion of reverse‐transported fog water utilized by these groups was 0%. Therefore, the analysis of reverse‐transported fog water utilization by *C. lanceolata* seedling leaves was conducted only for groups A–C. From group A to group C, the utilization of fog water by each part of *C. lanceolata* increased with the increase of fog duration (Figure 4), and the lower the SWC, the higher the proportion of stem and rhizosphere soils utilizing the reverse transported water when the duration of fog water environment was constant. At constant SWC, the longer the duration of the fog environment, the higher the proportion of leaves, stems, and rhizosphere soil utilizing water for reverse transport. That is, at 12 h, the maximum percentage of leaf, stem, and rhizosphere soil utilization of reverse transported fog water was 16.26% ± 1.21%, 11.13% ± 0.91%, and 1.66% ± 0.18%, respectively. The utilization of fog water by the leaves and stems was significantly higher (*p* < 0.05) than by the rhizosphere soil. The average utilization of fog water by the leaves, stems, and rhizosphere soil in groups A and B was 12.97%, 9.67%, and 1.11%, respectively. In contrast, no deuterium enrichment was observed in the rhizosphere soil of group C, resulting in a utilization rate of 0%.

## 4 Discussion

### 4.1 Characterization of Leaf Water Uptake and Reverse Transport

Generally, soil water migration occurs from the roots to the leaves along the water potential gradient (Hsiao and Xu 2000; Lee et al. 2018). Thus, LWP is closely associated with SWC and is directly influenced by precipitation (Liu, Yang, et al. 2021). In this study, leaf water absorption occurs when SWC falls below 60% of field capacity, indicating a threshold for soil and leaf moisture. The water potential in the leaf is consistently lower than that at the leaf surface, creating a reverse potential gradient that drives water absorption (Eller et al. 2013). Furthermore, when soil and leaf moisture levels are adequate, leaves show reduced sensitivity to precipitation, allowing plants to continue meeting their water needs primarily through soil moisture. Following mild soil drought, *C. lanceolata* is unable to absorb sufficient water, resulting in a state of water deficit in the leaves. This leads to a water potential that is lower than that of the atmosphere. Consequently, the water potential on the leaf surface exceeds that inside the leaves, creating a pressure gradient. Therefore, after 2 h of continuous wetting on the leaf surface, plants facilitate the movement of water into the leaves through stomata and other processes, leading to an enrichment of *δ*_*D*_ in the leaf tissues. This enriched water is then transported toward the stem and roots (Chen et al. 2022; Ellsworth et al. 2023). Additionally, due to the water deficit, the water potential in the roots and branches becomes lower than that in the leaves, creating a reverse water potential gradient among the three components. This gradient drives a reverse flow of water within the plant, moving it from the leaves downward to the roots, where it is eventually released into the soil (Hao et al. 2021). These findings are consistent with previous studies (Cassana et al. 2016; David et al. 2013). Furthermore, the results of this study indicate that when SWC falls below 45%–60% of field capacity, the *δ*_*D*_ values in leaves increase by approximately 20‰ as water is absorbed and transported reversely to the branches. When SWC is between 30%–45% of field capacity, water moves to the rhizosphere, resulting in an overall increase of about 30‰ in *δ*_*D*_ values. This suggests that under drought stress conditions, when field capacity is below 60%, the leaves not only utilize surface water to alleviate their own drought stress but also transport this absorbed water along the water potential gradients of the leaves, branches, and roots to the xylem and root systems over time (Fan et al. 2023; Gul et al. 2023; Liu, Zhang, et al. 2021). When soil moisture drops below 30%–45% of field capacity, the reverse movement of water to the rhizosphere results in reduced water availability. However, the increase in moisture significantly contributes to alleviating drought stress at the roots. This indicates that the leaf water absorption process is particularly sensitive to root drought pressure under extreme drought conditions, showcasing a more pronounced alleviation effect. Additionally, the ability of *C. lanceolata* to absorb water during short rainfall events may represent a strategic adaptation to arid environments.

### 4.2 Characteristics of the Proportion of Water Absorbed Through Leaf Water Uptake

Plants primarily rely on their root systems to absorb water for normal growth (Kang et al. 2022). However, some species can also absorb water through their leaves in arid environments to cope with drought stress, thereby improving water use efficiency (Farooq et al. 2019). The results of this study demonstrate that under drought stress, *C. lanceolata* can actively absorb intercepted precipitation through its leaves in foggy conditions, enhancing LWP. Following this water absorption, the leaves transport moisture downward through reverse water potential gradients to the branches and rhizosphere soil, alleviating the impact of drought. Additionally, the study indicates that the distribution strategy for fog water absorption by *C. lanceolata* varies under different soil moisture conditions and durations of fog events. As the duration of foggy conditions increases and SWC decreases, the phenomenon of FWU becomes more pronounced. Overall, under severe drought stress, plant leaves respond more quickly and sensitively to water availability. This heightened response may be attributed to the urgent water needs of the plants after significant water depletion. When precipitation occurs, the external moisture conditions exceed the LWP, facilitating a gradient‐driven continuum between the atmosphere, plants, and soil (Rattan et al. 2024). A faster circulation rate within this continuum leads to a greater volume of water transport, resulting in higher foliar utilization of surface rainfall. These findings are consistent with previous research (Feng et al. 2024).

Similarly, the longer the duration of foggy conditions, the higher the proportion of fog water utilization by *C. lanceolata* . In this study, the leaves and branches of the fir exhibited a higher utilization ratio of fog water, while the proportion near the rhizosphere was significantly lower. This discrepancy may be due to a portion of the absorbed water being temporarily stored in the leaves for photosynthesis, metabolic processes, and other uses (Hao et al. 2021). Another portion of the water is transported to the drier parts of the tree, where it is depleted through stomatal diffusion and transpiration, resulting in reduced water delivery to the branches and roots (Lee et al. 2018; Liu, Liu, et al. 2021). Additionally, after experiencing drought stress, the conductive structures used for water transport may be more susceptible to hydraulic conductivity loss due to xylem tension and air embolism (Allen et al. 2025; Liu et al. 2017; Pittermann et al. 2010), which could slow the reverse movement of water from the branches to the roots.

In summary, during the dry season, both air moisture content and soil moisture content are low. A portion of the water necessary for plant growth is derived from leaf absorption, while the remainder comes from root absorption. Given the high utilization rate of water absorbed by leaves in this period, their capacity to provide the necessary hydration for plants is enhanced, particularly when root replenishment is insufficient. Consequently, it is recommended that SWC should not fall below 60% of the field capacity during the dry season to adequately meet the water demands of plants. In the rainy season, both air moisture content and soil moisture content are high. In comparison to the dry season, the contribution of leaf water uptake to plant growth decreases, while root water uptake becomes more significant in supporting plant growth. Therefore, it is recommended to maintain soil moisture content at 60%–80% of field capacity during the rainy season to ensure an optimal soil moisture environment.

### 4.3 Limitations and Future Recommendations

This study highlights the significant role of FWU in alleviating short‐term drought stress. However, under prolonged water scarcity, the survival, growth, and physiological strategies of plants may become more complex. Future research could build on these findings by extending the experimental duration and incorporating relevant models to further illuminate the physiological trade‐offs and ecological adaptation mechanisms of plants in long‐term water‐deficient conditions. This approach will enhance our understanding of plant survival strategies in arid environments. Additionally, further studies should employ stable isotope labeling to investigate the conditions under which in situ growing plants absorb water and the ratio of precipitation that is utilized.

## 5 Conclusions

*C. lanceolata* effectively alleviates drought stress through FWU. In foggy conditions lasting for 2 h and when SWC is below 60% of field capacity, FWU occurs. If SWC is between 45% and 60%, water absorbed by the leaves reversely moves to the stem; when SWC drops to between 30% and 45%, the water further migrates to the rhizosphere soil. This indicates a heightened sensitivity to drought stress at the root level, leading to more pronounced alleviation effects. The utilization efficiency of fog water is higher in the leaves and branches; however, during the reverse water movement, a portion of water is gradually consumed, resulting in reduced quantities reaching the rhizosphere soil. Moreover, it is advised to maintain SWC above 60% during the dry season to meet the water demands of *C. lanceolata* . In contrast, during the wet season, SWC should be controlled between 60% and 80% to ensure healthy plant growth in optimal moisture conditions.

## Author Contributions

**Ting Xiang:** conceptualization (lead), data curation (lead), formal analysis (lead), methodology (equal), visualization (equal), writing – original draft (lead), writing – review and editing (lead). **Jianbo Jia:** conceptualization (supporting), funding acquisition (supporting), resources (supporting). **Bo Han:** investigation (supporting), methodology (supporting), supervision (supporting). **Chenhui Zhang:** software (supporting), visualization (supporting). **Wende Yan:** conceptualization (supporting), funding acquisition (supporting), resources (supporting).

## Funding

This research was supported by funds such as Key Research and Development Project of Hunan Province (2023SK2055), Natural Science Foundation General Program of Hunan (2023JJ31003), and the Hunan Water Conservancy Science and Technology Project (XSKJ2022068‐35; XSKJ2024064‐47). The authors gratefully acknowledge all financial support for this study.

## Conflicts of Interest

The authors declare no conflicts of interest.

## Supporting information


**Data S1:** ece372953‐sup‐0001‐DataS1.xlsx.

## Data Availability

All the required data are uploaded as Supporting Information.
